# Systematic optimization of ultrasound grayscale imaging presets and its application in abdominal scanning

**DOI:** 10.1002/acm2.13000

**Published:** 2020-08-11

**Authors:** Zaiyang Long, Wei Zhou, Donald J. Tradup, Scott F. Stekel, Matthew R. Callstrom, Nicholas J. Hangiandreou

**Affiliations:** ^1^ Department of Radiology Mayo Clinic Rochester MN USA; ^2^ Department of Radiology School of Medicine University of Colorado Anschutz Medical Campus Aurora CO USA

**Keywords:** grayscale, image preset, preset optimization, ultrasound

## Abstract

**Purpose:**

Ultrasound grayscale imaging preset optimization has often been qualitative and dependent upon vendor application specialists. This study aimed to propose a systematic approach for grayscale imaging preset optimization and apply the approach in a clinical abdominal scan setting.

**Methods:**

A six‐step approach was detailed including identification of clinical task, adjustment of basic parameters, fine‐tuning of advanced parameters, image performance metrics confirmation, clinical evaluation and data analysis, and implementation of new presets and monitoring of clinical usage. Its application in an abdominal scanning task was described for each step with phantoms, volunteers, and software tools.

**Results:**

Clinical image data analytics facilitated the understanding of the imaging task, relevant transducers, and target characteristics, in addition to specific requests from radiologists. Quantitative measurements were made on global image contrast and gray map function. In addition, clinically relevant phantoms and volunteer scans without and with acoustic distortion layers were involved to determine the new presets. Furthermore, phantom signal to noise ratio study and clinical evaluation using volunteers with different body habitus were utilized to confirm the superiority of the new presets. Quantitative clinical usage monitoring demonstrated successful implementation of the new presets.

**Conclusions:**

A systematic approach for grayscale imaging preset optimization has been proposed and successfully applied for a specific clinical task. This approach was designed to be generalizable and relatively flexible, which would facilitate movement away from previous qualitative and subjective approaches.

## 1 INTRODUCTION

It is well known that ultrasound imaging is more user‐dependent compared to other diagnostic imaging modalities. Part of the reason is that most acquisition parameters which fundamentally relate to image quality are frequently adjusted during scanning but cannot be changed after the fact. The images presented to radiologists may rely on features that sonographers have noticed and focused on. In addition, the bit depth of clinical ultrasound images is limited to 8 bits/pixel, that is, 256 shades of gray, compared to 10‐16 bits/pixel for other modalities. Consequently, radiologists do not have the capability to adjust display window setting to reveal additional information on different aspects of the anatomy because all the 8‐bit information are already being displayed on primary diagnostic monitors. Therefore, it is often relied on sonographers to have a wealth of clinical knowledge and scanning skills, who may also have to operate the scanner system under time pressure.

To facilitate clinical scanning, “presets,” which are predetermined sets of acquisition and processing parameters, are built on the scanners. They are often customized based on specific body parts, body habitus, transducers, and potentially clinical tasks, for example, performing a biopsy. Presets play an important role in ensuring image quality and clinical efficiency since they aim to greatly reduce the number of controls the sonographer must routinely adjust to optimize each image.^1^ New scanners generally arrive with a variety of factory default presets, followed by vendor application specialists working with clinical users to adjust them during clinical scanning based on the clinical needs and preferences. Traditionally, this process is qualitative and can be slow and highly dependent on the knowledge of the specific application specialist, the particular patients scanned, the image quality preferences of the sonographers and radiologists involved, and the communication process between the application specialist and the clinical practice.

In a modern radiology practice, it is common to have ultrasound systems of multiple software versions, multiple scanner models from one vendor, or even scanners from different vendors. Adjusting presets to ensure consistent image presentation and optimizing presets can be a complex task. For example, in our practice, patients who are scheduled for imaging‐guided ablation^2^ receive a pre‐ablation ultrasound examination for treatment planning purpose to assess the target, treatment angle, patient positioning, etc. Then, the ablation treatment starts with visualization of the target and placement of treatment applicators under ultrasound imaging guidance. High‐end ultrasound systems from two major vendors are involved in the diagnostic and interventional imaging guidance tasks in our practice, as described above. Despite the fact that quantitative phantom measurements of imaging metrics such as spatial resolution and contrast were comparable between the two systems,^3^ radiologists were not satisfied with the image appearance on one vendor system (denoted as system 1), and preferred the system of another vendor (denoted as system 2). Adjustment of image presets might solve the issue with system 1; however, there is a lack of study of preset optimization in the literature.^4^, ^5^

Therefore, the aim of this study was to establish a systematic approach for grayscale imaging preset optimization and apply it to optimize our abdominal presets on system 1 used in the abovementioned clinical setting.

## 2 MATERIALS AND METHODS

**Fig. 1:**
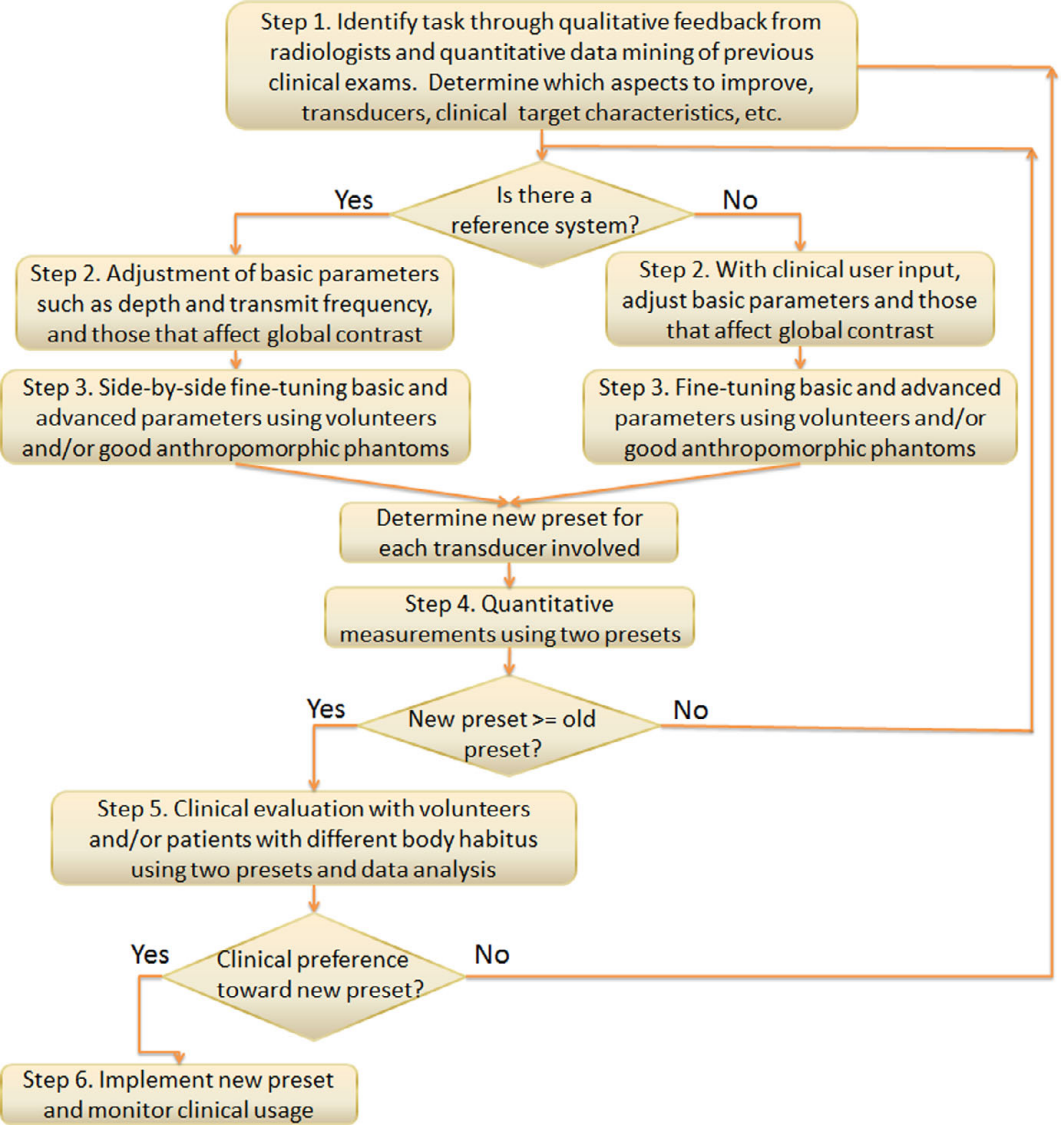
Flow chart for the proposed grayscale imaging preset optimization process.
  **Table 1:** The image views that were selected from an abdominal complete exam and used for the clinical evaluation with the accompanied questions

Our systematic approach is described in the following steps (Figure 1). Our application in this abdominal scanning setting is also included. Due to the initial quality improvement nature of this project and the nature of ultrasound scanning, this study did not require review from the local institutional review board.

1. Identification of the clinical task, that is, the aspects of image quality and/or appearance that need to be improved. Approaches could include qualitative feedback from radiologists in the clinical area, and quantitative data mining of previous clinical examinations. In reality, radiologists’ feedback could be as simple as “to make system 1 look closer to system 2,” that is, there is a reference system. Ideally, there would also be descriptions of what specific aspects of the images need to be improved with or without a reference system. This study will focus more heavily on the situation where there is a reference system. In our application of this step, clinical language and context were used to facilitate communication. Furthermore, data mining of previous clinical images was used to determine target characteristics such as echogenicity, depth, and size, as well as acquisition conditions such as commonly used transducers and transmit frequencies, which can be very helpful for determination of phantom targets and acquisition conditions for the next steps. An in‐house MATLAB program (MATLAB R2016, MathWorks Inc., Natick, MA) had been implemented for clinical informatics mining. It extracts information from clinical DICOM image header elements as well as information on the images through optical character recognition. ^6^
2. Adjustment of basic default/initial preset settings that might be changed frequently in clinical examinations to optimize patient images, such as imaging depth, gain, frequency, and transmit focus, as well as those which might be less frequently changed but can affect global image appearance, such as dynamic range, gray map, and tint map. Basic ultrasound phantoms and clinical image analytics were used. In our application of this step, initial preset settings on system 1, including depth, frequency range, and transmit focus, were checked with prior clinical data statistics and also compared with those from system 2. Gray map function was plotted using a custom MATLAB program in order to identify a map function on system 1 which would be close to system 2. A CIRS Model 040GSE phantom (CIRS Inc., Norfolk, VA) was used to measure global contrast during the parameter changes with the gray‐scale targets (−9, −6, −3, +3, +6 dB) using the UltraiQ software (Cablon Medical B.V., Leusden, The Netherlands),^7^ in order to confirm that preset changes were moving toward the right direction, that is, improving contrast as identified in step 1. If there is no reference system to try to move toward, radiologists and sonographers might need to be present or provide timely input, especially for subjective preferences such as tint map.
3. Fine‐tuning basic parameters and more advanced parameters such as spatial compounding, speckle reduction, and noise reduction, using volunteers and/or good anthropomorphic phantoms if volunteer scanning is not feasible. In our application, a CIRS Model 057A 3D abdominal phantom (CIRS Inc., Norfolk, VA) was utilized because of the abdominal imaging task. Additional custom‐made fat‐mimicking acoustic distortion layers (3‐6 cm thick) with specified speed of sound (1450 m/s), attenuation (0.6 dB/cm/MHz), and backscatter (0.003 sr^‐1^ cm^‐1^) properties were also utilized to make the phantom more realistic (CIRS Inc., Norfolk, VA). Because there was a reference system in our application, the two systems were set up in a side‐by‐side fashion equipped with comparable transducers of interest. Targets in the phantom such as the liver and vessels were utilized in the optimization task, for example, focusing on the targets while adjusting parameters to improve parenchyma texture, margin tightness, target contrast or noise in the vessels. Qualitative visual assessment was used under typical ultrasound scanning room illuminance level. In addition, volunteers were also scanned in a similar fashion first without and then with distortion layers for each transducer of interest. Subtle changes to basic parameters from step 2 might still be necessary because of real‐time scanning of the better test objects. Frame rate was also checked to make sure that the new presets did not have a significantly lower frame rate, before they were established and saved.
4. Image performance metrics confirmation of the new presets, which may include measurement of lesion signal noise ratio (SNR),^8^, ^9^, ^10^ resolution integral,^11^ or low‐contrast detectability.^12^ This step is to ensure that preset changes would not inadvertently lower the system performance. Here, 10 image pairs were acquired from the 4‐mm anechoic spherical targets (GAMMEX Model 408 spherical lesion phantom, GAMMEX Sun Nuclear, Middleton, WI) at 4‐8 cm of depth and from corresponding background regions using both the original and new presets on system 1. SNR values were computed and compared using a Wilcoxon signed‐ranks test.^13^ In order to be acceptable, the new preset needs to demonstrate comparable or better performance compared to the original preset.
5. Clinical evaluation and data analysis. In our application of this step, image views from the abdomen complete examination were reviewed and 17 out of 43 views were selected (Table 1). Each view was determined to correspond to a main underlying image quality metric to be asked in a reader study, such as spatial resolution, noise, and artifact. For example, noise limits the assessment of anechoic anatomies such as vessel lumens. The total number of views had to be reasonable for the readers to review in a relatively short amount of time. These specific views were acquired from three volunteers of differing body habitus using the original and new presets on system 1, with the imaging plane of each view closely matched. The body mass indices of the three volunteers were 21, 30, and 42. Images were then anonymized and information such as preset name and acquisition parameters was removed.

View name	Question
Longitudinal spleen	Which image is preferred for making an accurate spleen length measurement?
Longitudinal spleen/left kidney	Which image is preferred for assessing echotexture of the spleen compared with the kidney?
Longitudinal left kidney (pole to pole)	Which image is preferred for making an accurate kidney length measurement?
Longitudinal left kidney	Which image is preferred for assessing kidney anatomy?
Longitudinal proximal aorta	Which image is preferred for making an accurate aortic diameter measurement?
Longitudinal distal aorta	Which image is preferred for making an accurate aortic diameter measurement?
Transverse pancreas	Which image is preferred for visualizing the pancreas?
Longitudinal pancreas tail	Which image is preferred for visualizing the pancreas?
Longitudinal medial left lobe liver	Which image is preferred for evaluating the hepatic vein lumen in this normal subject?
Longitudinal caudate lobe liver	Which image is preferred for delineating the caudate lobe of the liver?
Transverse left liver/IVC	Which image is preferred for clear demonstration of the hepatic vessels?
Longitudinal right liver/right kidney	Which image is preferred for assessing the echotexture of the liver and kidney cortex?
Common hepatic duct	Which image is preferred for visualizing the hepatic artery and measuring the common hepatic duct?
Common bile duct	Which image is preferred for clearly visualizing the common bile duct as far as possible from the liver?
Longitudinal gallbladder decubitus supine	Which image is preferred for assessing the gall bladder lumen in this normal subject?
Transverse gallbladder decubitus supine	Which image is preferred for assessing the gall bladder wall?
Longitudinal right liver	Which image is preferred for assessing the liver contour?
  A total of 51 paired images acquired with the original and new presets were randomized and shown to a total of 14 readers including three radiologists who perform diagnostic imaging and ablation tasks, three ablation sonographers, three general sonographers with experiences less than 5 years, three sonographers with experiences longer than 10 years, and two sonographer educators. A specific question was asked with each pair of images, which was tied to the abovementioned quality metric associated with the image view (Table 1). For example, “Which image is preferred for evaluating the hepatic vein lumen in this normal subject?” The readers went through the evaluation on workstations in their typical work environment. Answers to all questions were collected and analyzed. The preference rate was calculated and compared between the two presets using a paired‐sample Wilcoxon signed‐rank test. Intraclass correlation coefficient (ICC) was computed to assess the agreement among the 14 readers
6. Implementation of new presets and monitoring of clinical usage. In our application of this step, the new presets were added on the scanners of the same vendor and model as system 1, while keeping the original presets so clinical users can choose during clinical scanning. Sonographers were notified about the new presets. The in‐house MATLAB clinical informatics program was used to monitor the preset usage in the following 120 days after implementation, to assess the success level of the preset optimization. The percentage of usage of the new preset was calculated for each transducer involved. A higher percentage compared to that of the original preset would be considered as successful. Otherwise, the reason would be further investigated.

## 3 RESULTS

From step 1, the clinical task in our application was to adjust the image appearance from system 1 to be closer to system 2, including to enhance image and target contrast, decrease artifact and noise in theoretically anechoic targets (such as simple cysts), and improve edge sharpness. Prior clinical examination statistics revealed that the lesions that were clinically relevant were>=1 cm in diameter with the majority of the depths ranging 4‐8 cm. Three transducers of system 1 were identified for improvement for this abdominal scanning task including two curvilinear and one linear. Results from the most commonly used curvilinear probe will be focused here.

**Fig. 2:**
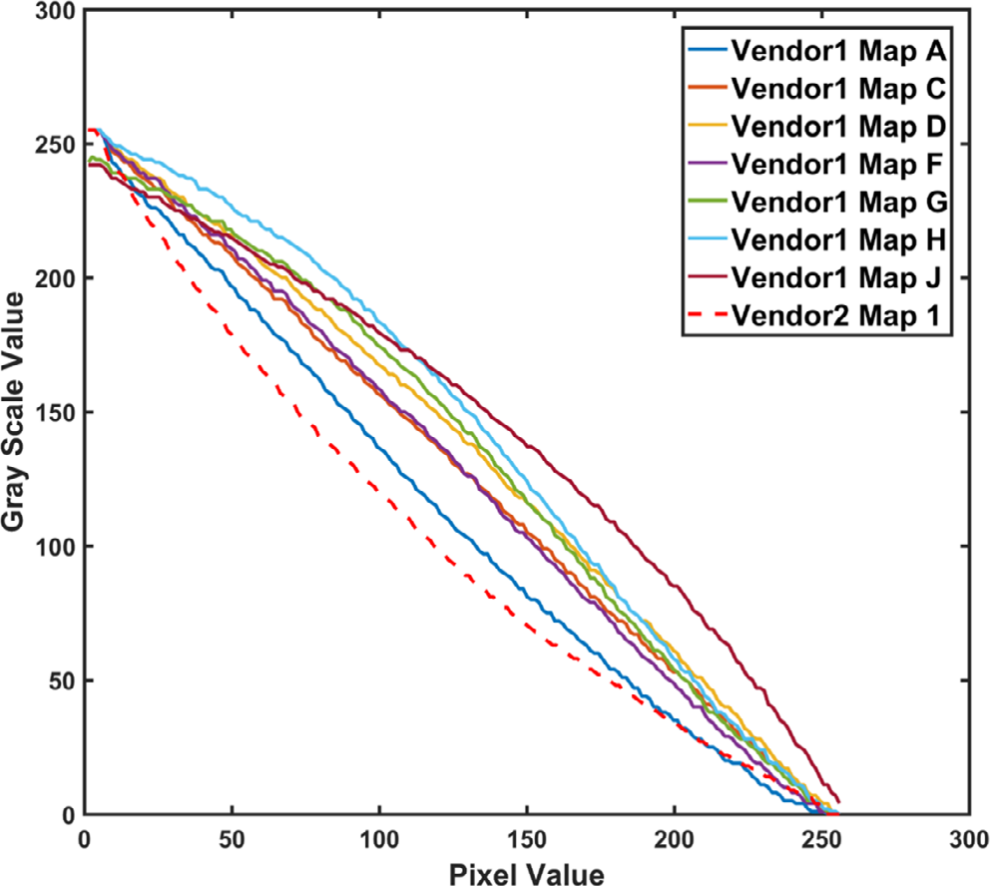
Gray map function options for a curvilinear probe on system 1 compared to the map for the similar probe on system 2. Gray map A was consequently selected on system 1.

**Fig. 3:**
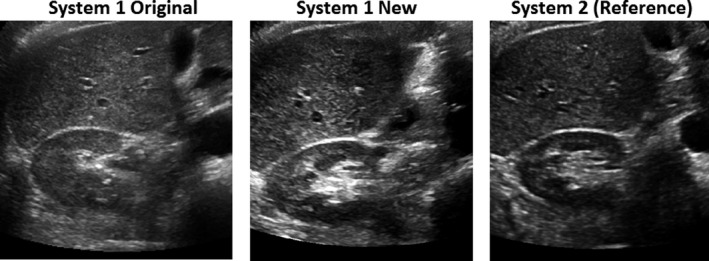
Examples of volunteer images of the same view acquired on both systems during step 3 of fine‐tuning parameters and determination of the new preset.

From steps 2 and 3, a new preset was established with updated parameters through prior clinical data analytics and side‐by‐side adjustment of acquisition parameters with the reference system, using phantoms and volunteers without and with acoustic distortion layers. For example, dynamic range was decreased (from 69 to 51), speckle reduction imaging parameter increased (from 1 to 3), rejection increased (from 0 to 1), and the closest gray map function was selected. Examples of gray map function options were plotted for both systems (Figure 2). The mean global contrast was shown to be 5.2 and 6.6 gray levels/dB for the original and the new preset on system 1, respectively. It was 4.7 gray levels/dB on the reference system. An example of volunteer images using the original preset, new preset, and reference system during parameter adjustment is shown in Figure 3.

**Fig. 4:**
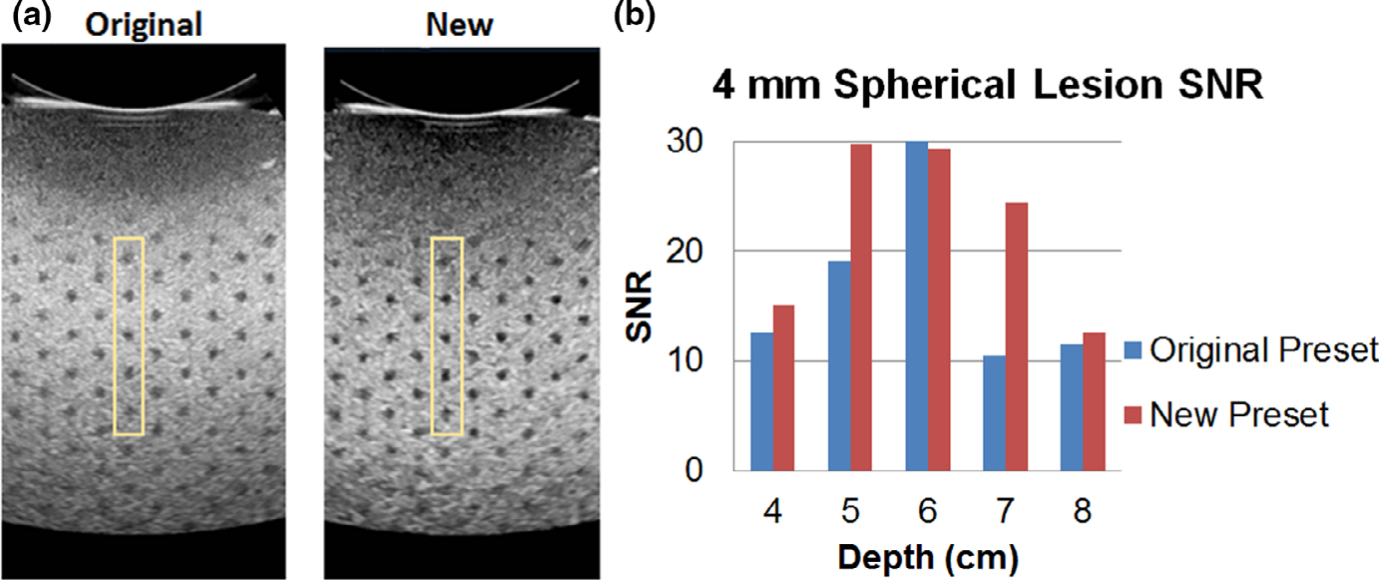
Signal‐to‐noise ratio measurements with a pair of example phantom images (a) and results (b) from 4‐mm anechoic targets to verify that the new preset would perform at least as well as the original preset during step 4.

Furthermore, in step 4, lesion SNR measurements were obtained at target depths of 4‐8 cm. An example of the phantom images acquired with the two presets is demonstrated in Figure 4. SNR results from the new preset were demonstrated to be significantly higher than those from the original preset (p < 0.05).

**Fig. 5:**
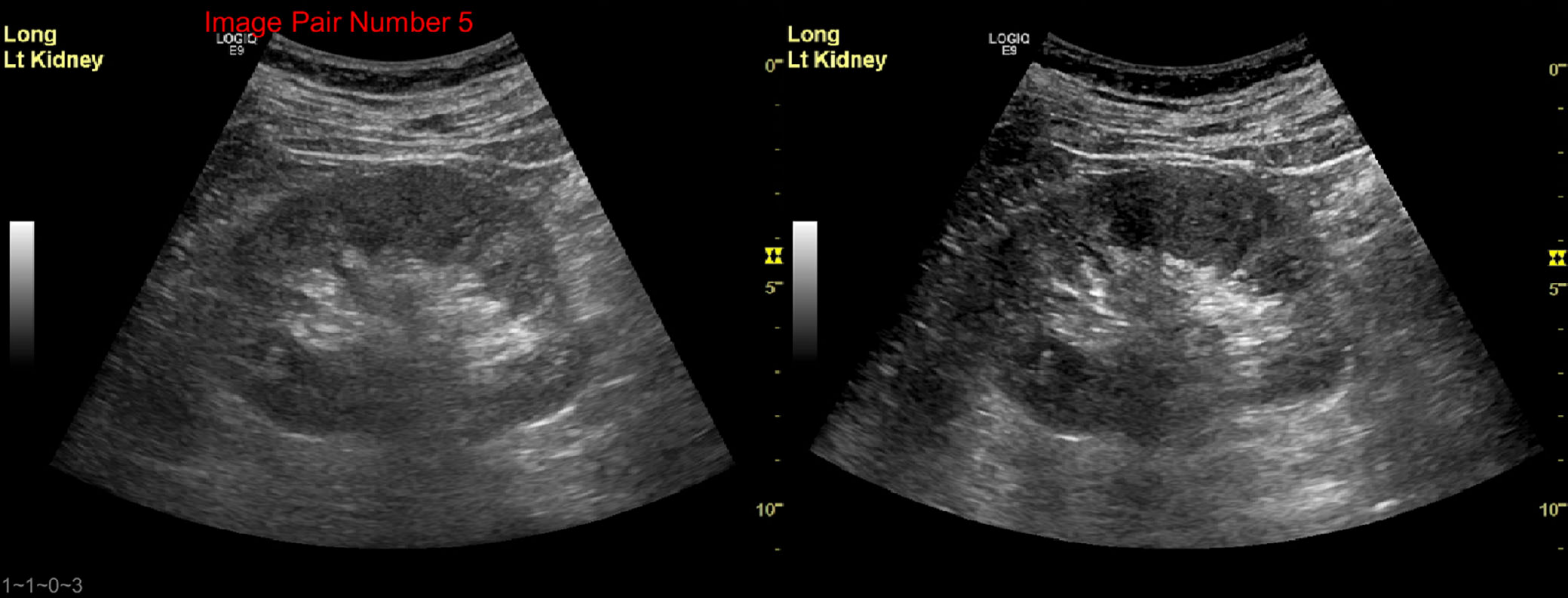
Example of a volunteer image comparison between the original and new presets with the question “Which image is preferred for making an accurate kidney length measurement?” during clinical evaluation in step 5.

**Fig. 6:**
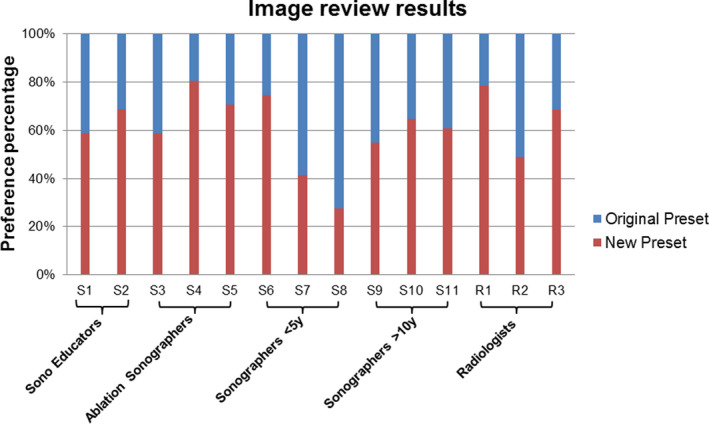
Clinical evaluation results shown in preference percentages between the two presets from 14 readers, including two sonographer educators, three ablation sonographers, three sonographers with < 5 yr of experience, three sonographers with more than 10 yr of experience, as well as three radiologists.

For the clinical evaluation in step 5, an example of the questions is shown in Figure 5, including a pair of volunteer images for a longitudinal kidney view acquired with the two presets, together with a question of “which image is preferred for making an accurate kidney length measurement.” An average of 61.2% preference toward the new preset was demonstrated among all readers and also shown to be statistically significant (p < 0.05; Figure 6). ICC with 95% confidence interval (CI) was 0.792 (95% CI 0.697‐0.867) among the 14 readers (p < 0.001), indicating good reliability.

In the 120 days after implementation of the new preset in step 6, 77.9% of the total 1343 images were acquired using the new presets, including 76.8% and 92.2% for the two curvilinear transducers, and 70.1% for the linear transducer.

## 4 DISCUSSION

In this study, a systematic approach was proposed for ultrasound grayscale imaging preset optimization. This approach was successfully applied to an abdominal preset optimization task. Clinical image data analytics is a very useful aid to identify the imaging task, target characteristics, and monitoring the effects of preset changes. Clinically relevant phantom and volunteer scans without and with additional acoustic distortion layers were involved to determine the new presets. Furthermore, phantom SNR study and clinical evaluation using volunteers with different body habitus were utilized to confirm the superiority of the new presets over the original presets. Quantitative clinical usage monitoring, as mentioned above, demonstrated successful implementation of the new presets. Positive verbal feedbacks on the new presets were also received from clinical users. Optimized presets would not only aid in radiologists’ clinical tasks, but also increase sonographers’ efficiency by shortening the time they had to spend on manipulating acquisition and processing parameters.

The parameters changed for the specific abdominal task corresponded to the clinical request. A moderate decrease in dynamic range increases image contrast. Dynamic ranges too low or too high will result in loss of information or a very flat image. An automatically adjustable, region of interest‐based approach on dynamic range would be beneficial.^14^ Furthermore, global contrast measurements were performed to confirm the increased contrast using the new preset. It is worth noting that global contrast measurements reflected vendor differences in image acquisition and processing, and the results were not meant to directly match between vendors. Speckle reduction and suppression improve image noise performance and edge delineation. In addition, the gray map function was changed to be closer to that on the reference system. The acoustic distortion layers to make the scanning more realistic and volunteers with a wide range of body habitus are essential to ensure high clinical relevance of this process.

In addition, default imaging depth, frequency range, and focus on system 1 were checked against prior clinical analytics data and the reference system. It is worth noting that the reference system may not be the ultimate target for optimization. For example, if clinical data statistics revealed a very different imaging depth or gain compared to the default start on the reference system, these parameters should be updated to reflect the clinical data statistics, which would also shorten the time sonographers needed to change the parameters when scanning on the reference system. To facilitate grayscale optimization work in general, Table 2 shows possible parameters for adjustment for a variety of tasks as a reference point.

**Table 2:** Potential parameters for adjustment for a variety of imaging aspects and tasks as a reference

Contrast and presentation	In‐plane spatial resolution	Speckle	Noise	Posterior Artifacts	Needle visualization
Dynamic range, gray map, tint map, harmonic imaging	Transmit frequency, transmit focus/focal zone, harmonic imaging, line density, tissue aberration correction (if available)	Spatial compounding, speckle reduction	Spatial compounding, noise suppression, rejection	Spatial compounding, transmit focus/focal zone	Beam steering, spatial compounding, dedicated needle visualization tools (if available)

Additional notes that we believe to be useful are the following: Understanding the vendor‐specific control parameters and having a person with good clinical scanning skills on the team really facilitatethis type of work. A vendor application specialist could also assist in these regard. Utilizing a side‐by‐side scanning configuration for immediate and direct comparison of new images with those from the original preset or from a reference system is also helpful. This approach also allows image pairs to be consistently acquired in the same imaging plane for clinical user feedback. When adjusting controls that alter frame rate, it is important to make sure the frame rate in the new configuration is not considerably lower than that in the original preset. Last but not least, since many tasks were evaluated on the scanner display monitor, the monitor should be calibrated according to DICOM Gray Scale Display Function (GSDF) to ensure visual consistency with PACS workstations.^15^ Both system monitors involved in the study were calibrated according to DICOM GSDF. If either system display is not, one should explore the possibility of enabling DICOM GSDF, or care should be exercised to ensure image appearance consistency with PACS workstations. Finally, it is clear that reader preference played a role in the clinical image review results. Out of the 14 readers, two sonographers with less than 5 years of experience preferred the original preset over the new preset and one radiologist rated them about the same. When evaluating images, it is important to have multiple readers or at least the main stakeholders.

Protocol management and optimization contributes significantly to the delivery of meaningful patient care.^16^ However, it is still often lacking in ultrasound in comparison to some other modalities. In addition, presets can be modified relatively easily on the scanner system; therefore, they can evolve unintentionally if there is no close monitoring. Therefore, differences in preset settings among scanners of the same vendor and model, as well as the variations in image appearance are not rare. Sometimes, a clinical request for improved image quality might be met by harmonizing these presets on scanners of the same vendor and model. A computerized tool that can compare, monitor, and distribute presets on various scanners would be highly useful. Together, these efforts could result in significant improvement of ultrasound preset management and optimization.

This study has a number of limitations. First, the proposed optimization approach involved a number of steps and various phantoms or tools, and was applied in only one clinical setting. However, the approach itself was designed to be generalizable and flexible. Phantoms and acoustic distortion layers utilized in parameter adjustment may not be readily available for some readers, or not applicable for other types of clinical tasks. Nevertheless, images from volunteers and/or patients with different body habitus can always offer alternative approaches. Second, commercial phantom targets are close to but not perfectly match the clinical target in this task. For example, prior clinical patient statistics revealed that the lesions of interest were>=1 cm in diameter for this task. However, 4‐mm anechoic targets were the largest in the Gammex phantom; therefore, these were imaged for SNR measurements. Consequently, SNR results were all well above the visual detection threshold. Nevertheless, these results provide a comparison between the two presets to confirm the new preset does not inadvertently perform inferiorly to the original. Third, ideally the clinical evaluation study would have only been performed on workstations with DICOM GSDF calibrated monitors. Due to the time constraints and availability, this was not the case for the sonographers. They used their workstations equipped with HP EliteDisplay E243i monitors (HP Inc., Palo Alto, CA) for clinical image viewing and quality control in their daily workflow. Visual assessment of the AAPM TG18 multipurpose pattern on these display monitors showed that the 5% and 95% patches were visible. ^15^

## 5 CONCLUSION

In conclusion, we have proposed a systematic approach for grayscale ultrasound imaging preset optimization. This approach was successfully applied in a specific abdominal imaging task, where new presets were generated, evaluated, implemented, and monitored clinically. The approach was designed to be generalizable and relatively flexible, which would facilitate movement away from previous qualitative and subjective approaches.
